# Bilateral giant parathyroid adenoma and hungry bone syndrome: a case report

**DOI:** 10.1186/s13256-023-04102-w

**Published:** 2023-09-01

**Authors:** Carolina Martínez-Loya, Dalai E. Granados-Gutiérrez, Anagabriela Torrens-Chacón, David A. Rodríguez-Luna, Zabdy E. Frayre-García, Leonela Villegas-Vázquez, Luis B. Enríquez-Sánchez

**Affiliations:** 1 Departamento de Investigación, Universidad Autonóma de Chihuahua, Facultad de Medicina y Ciencias Biomédicas, Nuevo Campus Universitario, 31125 Chihuahua, Mexico; 2 Departamento de Cirugía, Hospital Central del Estado, Antonio Rosales 33000, Obrera, 31350 Chihuahua, Mexico; 3 Departamento de Medicina Interna, Hospital Central del Estado, Antonio Rosales 33000, Obrera, 31350 Chihuahua, Mexico; 4 Médico Interno de Pregrado, Hospital Central del Estado, Antonio Rosales 33000, Obrera, 31350 Chihuahua, Mexico

**Keywords:** Bilateral parathyroid adenoma, Primary hyperparathyroidism, Hypercalcemia

## Abstract

**Background:**

There is some evidence supporting the idea that double parathyroid adenomas represent a different entity from multiglandular hyperplasia; however, the distinction among them is not straightforward.

**Case presentation:**

We described a case of primary hyperparathyroidism (PHPT) with pronounced clinical manifestations, caused by a bilateral giant parathyroid adenoma. A 34-year-old Hispanic/Latino male was diagnosed with PHPT caused by two giant parathyroid adenomas. The preoperative tests were neck ultrasound and computed tomography scan (CT-scan), showing two masses in the territory of parathyroid glands, bilaterally distributed (right was 31 × 18 × 19 mm and the left was 38 × 15 × 14 mm); sestamibi scan was not available. Parathyroid hormone (PTH) was highly elevated. Multiple complications of PHPT were present, such as bone lytic lesions, renal and pancreatic calcifications, and cardiovascular disease, the latter of which is an overlooked complication of PHPT. Multiple endocrine neoplasia 1 and 2 (MEN 1/2) were ruled out by the absence of clinical, biochemical, and radiological findings in other endocrine glands. The patient underwent subtotal parathyroidectomy with an intraoperative histopathological study; both intraoperative and definitive histopathology results were consistent with parathyroid adenomas; afterward, adequate suppression of PTH was assured, and later on, the patient presented hungry bone syndrome (HBS).

**Conclusions:**

The diagnosis of double parathyroid adenomas is difficult. Regarding the similarities between multiglandular hyperplasia and parathyroid adenomas, this case report contributes to the further distinction between these two clinical entities. This case report also represents, in particular, the challenge of difficult diagnosis in places with limited resources, such as developing countries.

## Introduction/background

PHPT is an endocrine disorder characterized by excessive secretion of PTH, which can be secreted by one or more of the four parathyroid glands. PHPT is commonly caused by a single benign parathyroid adenoma [1], and excessive PTH leads to an increase in blood calcium. PHPT was first described approximately 90 years ago and has evolved from a severe disease characterized by the expression “stones, bones, and groans” (which refers to clinical manifestations of urolithiasis, osteopenia, and gastrointestinal disturbances, respectively) to a generally asymptomatic entity that is discovered fortuitously [2]. In most cases (80%), PHPT is caused by a single adenoma of one of the parathyroid glands; 10 to 11% of patients have more than one adenoma; another 10% have four-glands hyperplasia, and less than 1% of PHPT cases are caused by parathyroid carcinoma [3]. In recent years, it has been proposed that double adenomas represent a different clinical entity, and they should not be confused with an asynchronous form of multiglandular hyperplasia [4]. A PHTP diagnosis is established when there is hypercalcemia accompanied by inappropriate PTH suppression (values are normal or high). Moreover, 25-hydroxyvitamin D levels may be normal or low-normal in patients with PHTP, and 1,25-hydroxyvitamin D is usually elevated or normal-elevated [5]. Parathyroidectomy remains the only definitive cure and is the most cost-effective treatment. Surgery is highly successful, with cure rates ranging from 94 to 99% [6]. Giant parathyroid adenomas (GPAs) are defined as weighing more than 3 grams, and the presence of bilateral parathyroid adenomas in the absence of multiple endocrine neoplasia 1 (MEN 1) is extremely rare [7]. In this report, we discuss the case of a patient with two GPAs, bilaterally distributed (9 and 7 gr), with classic clinical manifestations of PHPT, in which the presence of MEN 1/2 was discarded by the absence of clinical, biochemical, and radiological findings in other endocrine glands (pituitary gland, pancreas, thyroid gland, suprarenal glands).

## Case history

**Fig. 1:**
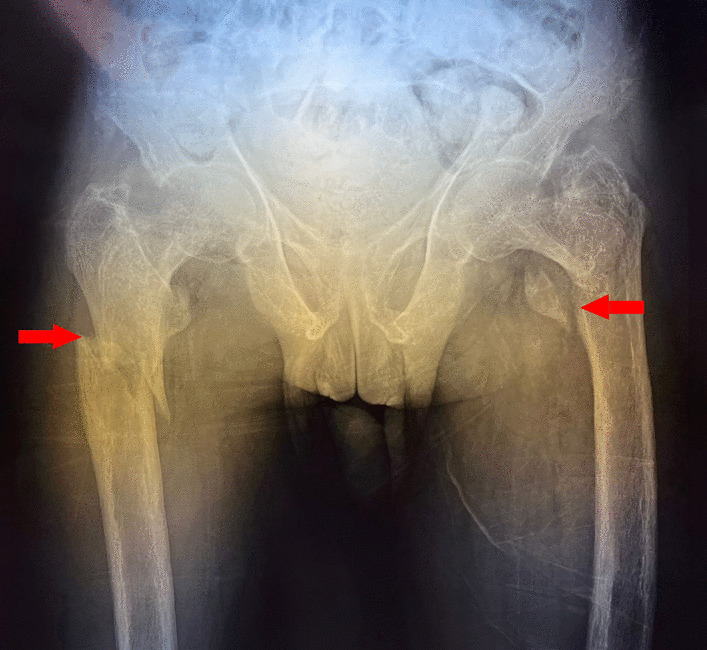
Pathological fractures. Right subtrochanteric femur fracture (right left arrow) and left pertrochanteric femur fracture (left red arrow)

**Fig. 2:**
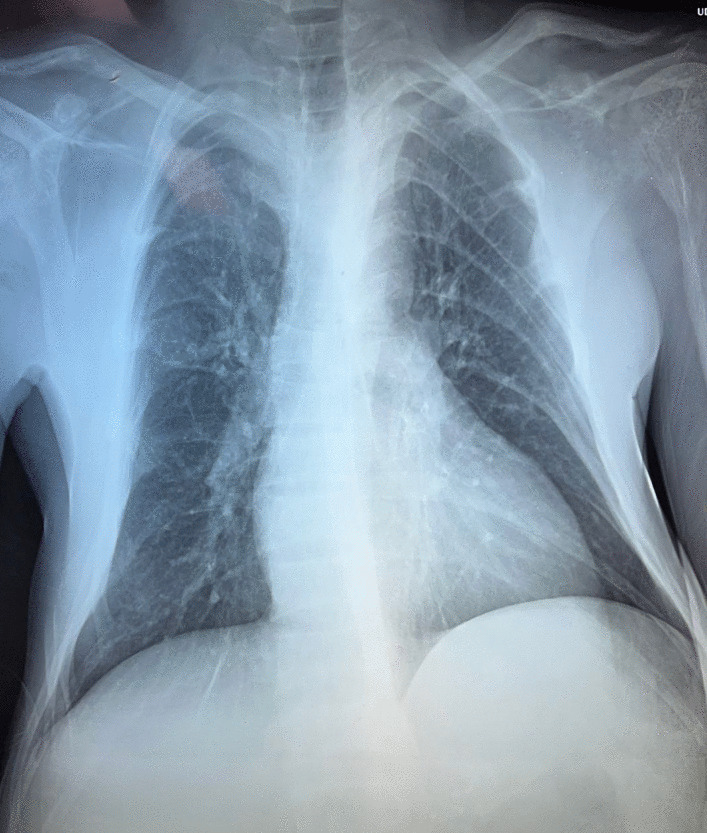
Beaded ribs. Expansion of the costochondral junctions

**Fig. 3:**
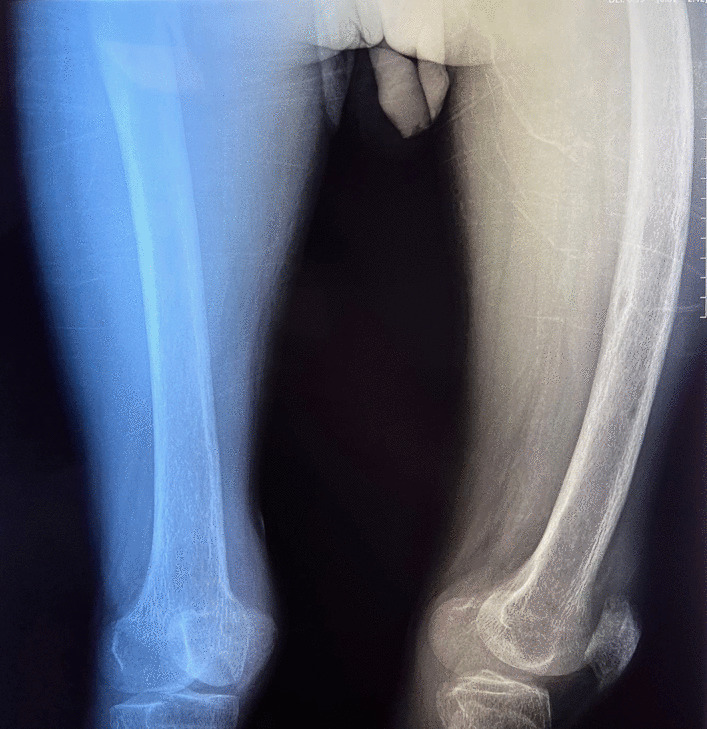
Cortical bone thinning. The right femur is internally rotated and the left is externally rotated

**Fig. 4:**
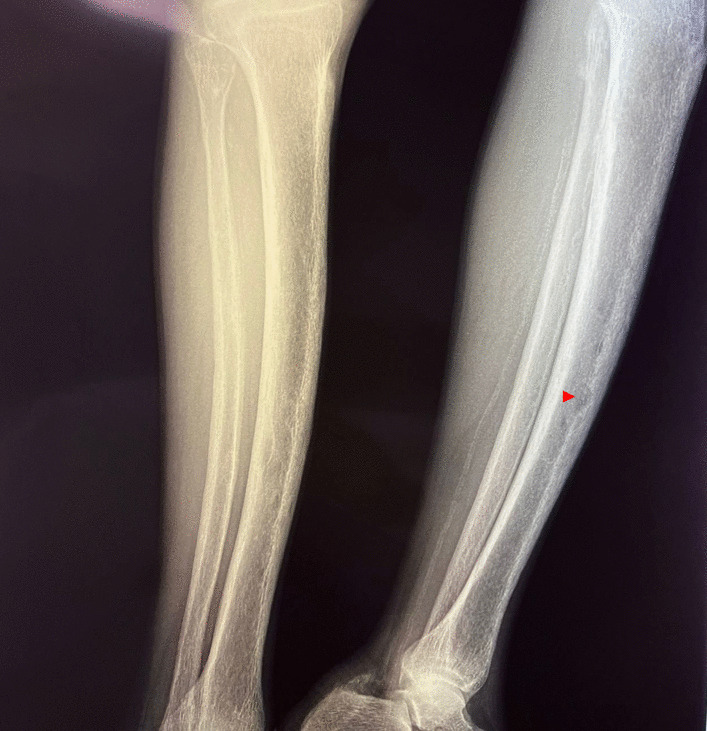
X-ray of the tibia and fibula showed lytic lesion (arrowhead)

**Fig. 5:**
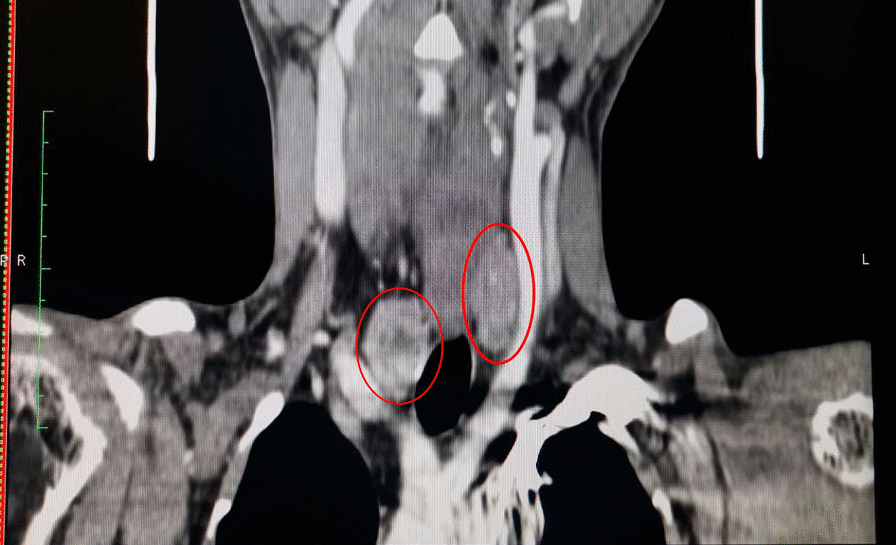
Computed tomography of the neck with IV contrast. Bilateral heterogeneous nodules in the region of the parathyroid glands (red ovals)

**Fig. 6:**
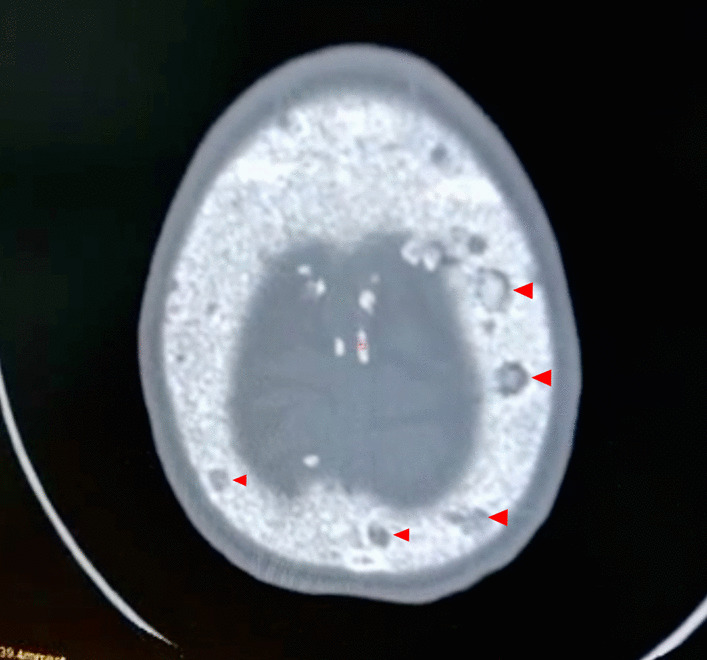
Skull CT. Lytic lesions in the skull bone (arrowheads)

**Fig. 7:**
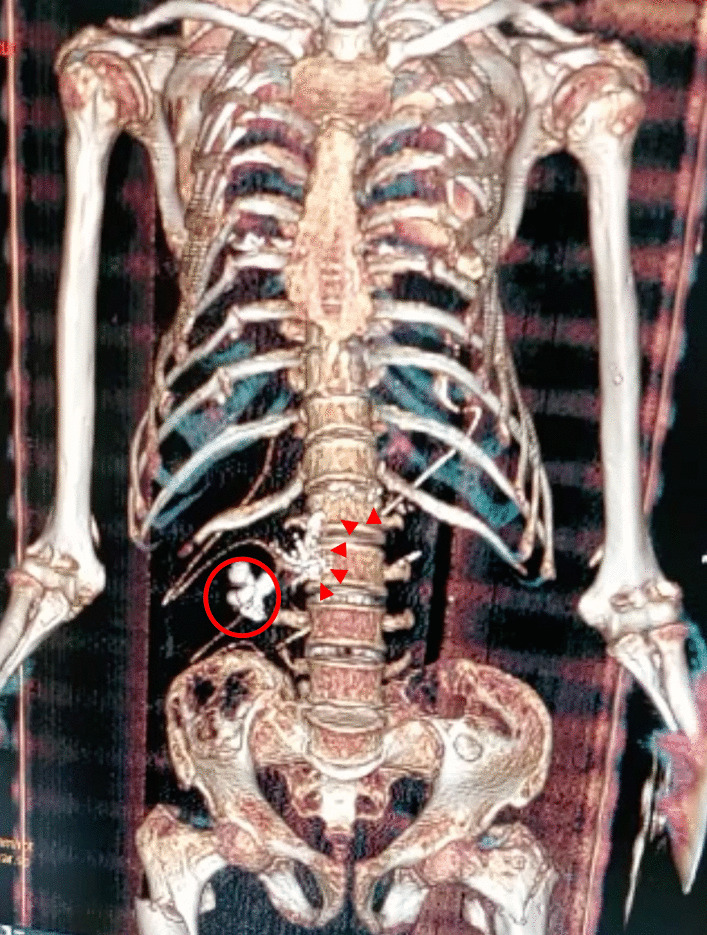
CT 3D bone reconstruction. Staghorn calculus (red circle) and pancreatic calcifications (arrowheads)

**Fig. 8:**
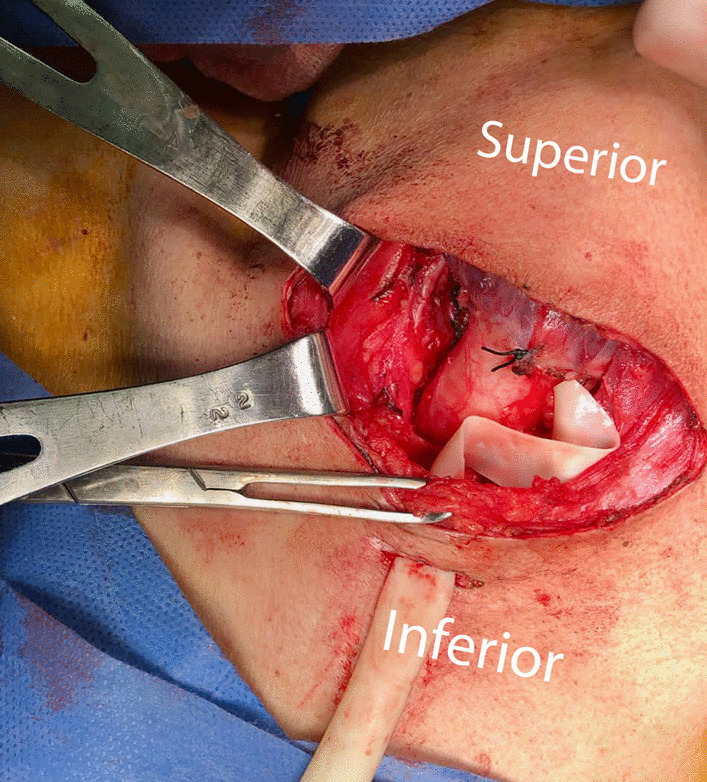
Surgical approach

**Fig. 9:**
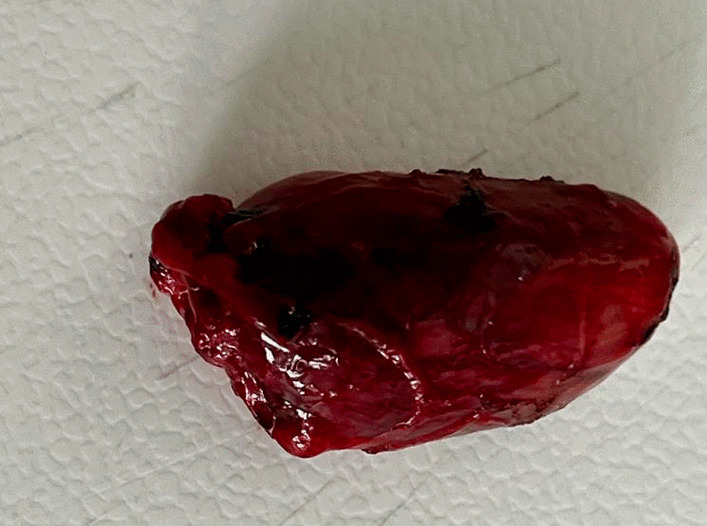
Excised right parathyroid tumor measuring 3.9 × 2.5 × 2 cm

**Fig. 10:**
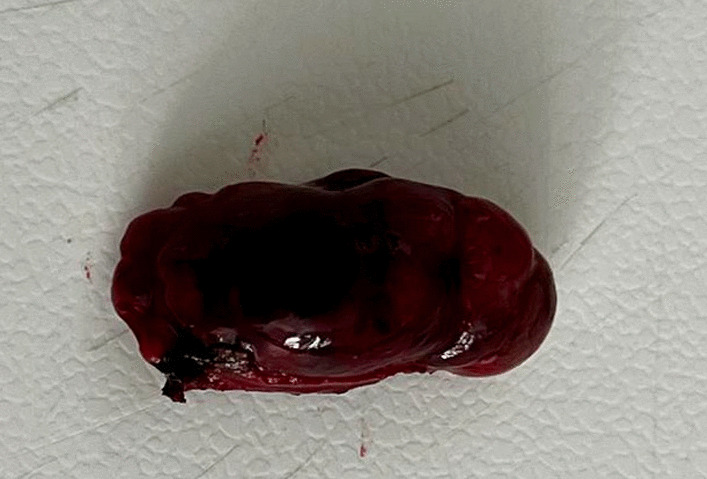
Excised left parathyroid tumor measuring 3.8 × 2.3 × 2 cm

A 34-year-old Hispanic/Latino male from a marginalized community suffered a fall from his own height. He reported pain in both hips and impaired gait; due to this, he went to a rural health center, where they identified bilateral hip fractures, consisting of a right subtrochanteric femur fracture and a left pertrochanteric femur fracture (Fig. 1). Physical examination showed right inferior limb with internal rotation, shortening of the left inferior limb with external rotation, and sharp sarcopenia in both inferior limbs. The patient presented muscle weakness, so the patient had used crutches to support himself in walking in the previous months. For most of his life, the patient lived in the Tarahumara region (a rural zone) and worked at a sawmill. Currently unemployed because of health issues. His highest educational level was high school. He used to smoke cigarettes (a pack every week since he was 16 years old), consume cocaine occasionally, and drink alcohol (every weekend getting drunk) but stopped consuming all these substances four months before the current illness. His prior clinical history included weight loss of 20 kg in a period of 5 years, chronic musculoskeletal pain, fatigue, debility, progressive elevation of serum creatinine and nitrogen compounds, and chronic elevation in alkaline phosphatase. The patient denied the consumption of any medication. At the presentation moment, the patient weighed 45 kg, his height was 160 cm, his temperature was 36.9 ºC, his blood pressure was 116 over 74 mmHg (mean 88 mmHg), respiratory rate 20 bpm, heart rate 99 bpm, oxygen blood saturation was 94%. The laboratory findings were leucocytes in 7.63 k cel/μL, hemoglobin in 6.3 g/dL with MCV in 105.8 fL, MCH in 33 mg/dL, and platelets 169 k/μL. Serum creatinine was 2.31 mg/dL, urea was 83 mg/dL, protein C reactive was 15.6 mg/L, sodium was 138 mEq/L and potassium was 5.9 mEq/L. He was transferred to our Central Hospital in the state of Chihuahua, Mexico, for his examination and management where laboratory exams were done, finding blood calcium increased, resulting in 13 mg/dL (normal 8.8–10.5 mg/dL), urine calcium was also elevated, resulting in 49.9 mmol (normal 2.5–7.5 mmol), and alkaline phosphatase (AP) was also highly elevated, resulting in 2126 IU/L (normal 44–147 IU/L). From the suspicion of PHPT as a result of the hypercalcemia and the pathological fractures, measurements for PTH levels were performed, resulting in 3000 pg/mL (normal range is 10–55 pg/mL). Levels of 25-hydroxyvitamin D were also measured, resulting in 5.7 ng/mL, thus evidencing a clear deficiency. High flow of intravenous (IV) crystalloid fluids (Hartmann 250 ml/hour) and furosemide (40 mg IV every 8 hours) were initiated in order to diminish the hypercalcemia. Analgesic measures with a tramadol IV infusion (200 mg in 100 ml with an infusion speed of 4 ml/hour) and acetaminophen (1 gr IV 3 times daily) were performed. Prophylaxis for venous thromboembolism was also initiated with enoxaparin (40 mg subcutaneously every 24 hours). Neck palpation was negative for adenopathies or masses. Full-body X-ray series were done in the chest, pelvis, femur, knee, tibia, and fibula, showing radiological signs such as moth-eaten pattern, lytic bone lesions, and general cortical bone thinning (Figs. 2, 3, and 4). A neck ultrasound was performed, finding two solid heterogeneous masses with perinodal and intranodal vascularity inferior to the thyroid gland; the right mass had dimensions of 31 × 18 × 19 mm, and the left was 38 × 15 × 14 mm. Afterward, a neck CT scan with intravenous contrast was performed to dictate surgical treatment, where two heterogeneous nodules in the territory of the superior parathyroid glands were reported (Fig. 5). It was not possible to realize the sestamibi parathyroid scan or bone gammagraphy scan because of limited resources in the hospital. A full-body CT scan was done to assess the presence of other tumors, and it demonstrated no disease in other endocrine glands (pituitary gland, pancreas, thyroid gland, and suprarenal glands). The genetic exam for NEM 1/2 was not available, although familiar history was negative for endocrine disorders. The full-body CT scan showed multiple bone skull lesions with rounded and heterogeneous aspects, apparently compatible with lytic lesions (Fig. 6). The 3D reconstruction evidenced numerous lytic lesions in multiple bones, numerous pancreatic calcifications, and a right staghorn calculus (Fig 7). An electrocardiogram showed left ventricular hypertrophy (LVH) using Sokolow-Lyon criteria, indicating heart affection secondary to the PHPT. Surgical treatment was the therapeutic decision, with parathyroid removal and an intraoperative histopathological study of both pieces (Fig. 8). Prior to surgery, calcitriol prophylaxis was initiated (0.5 mcg orally 3 times daily for 3 days), and calcium carbonate (500 mg orally per day) During surgery, the presence of a tumor in the right inferior parathyroid gland was identified and resected; the dimensions were 3.9 × 2.5 × 2 cm and 9 gr (Fig. 9). A left inferior parathyroid gland resection was performed, and the dimensions were 3.8 × 2.3 × 2 cm and 7 gr (Fig. 10). The intraoperative histopathologic study reported both pieces as parathyroid adenomas without any features of malignancy, yet parathyroid hyperplasia must be discarded. The definitive study results were the following: The right resected parathyroid gland was reported as a parathyroid adenoma, and the left resected parathyroid gland was reported as a parathyroid adenoma, hence, the existence of a bilateral parathyroid adenoma was concluded. Immediately after the procedure, calcium levels in serum were 9.4 mg/dL, and 2 days later, the calcium levels descended to 8.7 mg/dL, both results being corrected for hypoalbuminemia. In the following days, calcium in serum levels continued descending, so a calcium gluconate infusion had to be started, and the calcitriol dose was also increased, Concomitantly, phosphorus, and magnesium blood levels also went down. The patient was discharged 23 days after this surgery when serum electrolytes were stabilized, and the calcium gluconate infusion was withdrawn. Ambulatory treatment was prescribed with calcitriol 0.5 mcg every 3 hours orally, calcium carbonate 1 gram every 6 hours orally, and anticoagulation prophylaxis with rivaroxaban 10 mg every 24 hours. In the same admission, hip, and femur fracture correction was performed through closed reduction-internal fixation under the biomechanical principle of support with a cephalomedullary nail.

One month later in outpatient care, control levels of PTH and serum calcium were performed resulting in 459 pg/mL and 7.2 mg/dL respectively, treatment with calcium carbonate 500 mg orally every 8 hours, and calcitriol 0.25 mcg orally every 6 hours both continued for 3 more months. Six months later PTH and serum calcium were at 72 pg/mL and 9.4 mg/dL respectively, calcitriol dose was increased to 0.5 mcg every 6 hours, and calcium carbonate 500 mg every 6 hours. The patient also presented multiple renal calculous and nephrocalcinosis that caused renal pathology (serum creatinine 2.8 mg/dL and CDK-EPI 28 ml/min/1.73 m^2^) and transferred to nephrology. After 8 months calcium supplements and calcitriol were suspended.

## Discussion

Giant bilateral parathyroid adenoma presentations are rare and the complications of PHPT are hardly seen nowadays. The radiological findings of the present case report are the perfect example of severe complications of PHPT, this case report shares the natural history of the disease of PHPT which is not commonly found in the literature.

Parathyroid adenomas usually measure less than 2 centimeters and weigh less than 1 gram, GPAs are defined as weighing more than 3–3.5 gr, and they appear with PHPT syndrome [8]. In this case report, two GPAs (9 and 7 gr) were found, and the diagnosis was made through histopathology. In a historical review of PHPT, two stages are described: the first with pronounced clinical manifestations, such as bone disease, urolithiasis, and gastrointestinal symptoms, the second, founded more recently, is presented as asymptomatic individuals with mild hypercalcemia [9]; the latter is due to the increment of health services coverage and screening. This report presents a case of a patient with pronounced clinical manifestations, which is probably due to the long evolution of the PTH excess and the hypercalcemia because of the minimal access that the patient had to health services. At the time of the diagnosis, bone disease, kidney stones, and cardiovascular disease were present, the latter a forgotten complication of PHPT. The patient presented progressive mobility loss, radiological signs of osteoporosis, a bilateral pathological hip fracture, multiple rib fractures, and lytic lesions in numerous bones. The CT scan (Fig. 7) showed lytic lesions with a heterogeneous cystic component compatible with osteitis fibrosa cystica, a forgotten radiological feature of PHPT by the rare incidence of bone disease in PHPT [10], these lesions can be easily mistaken for bone metastatic disease [11]. Another PHPT manifestation is urolithiasis, which was encountered through the CT scan: demonstrating the presence of a staghorn calculus in the right renal pelvis. Additionally, multiple pancreatic calcifications can also be present in PHPT [12]. If these calcifications are present, they can cause pancreatitis by obstruction of the pancreatic ducts; moreover, hypercalcemia can lead to the de novo conversion of trypsinogen to trypsin [13]. Furthermore, the patient had LVH, and the prevalence of LVH is higher in PHPT. Cardiovascular risk increases in patients with PHPT, but tends to diminish after parathyroidectomy [14, 15]. Following the parathyroidectomy, the patient presented hypocalcemia, hypophosphatemia, and hypomagnesemia, all of which persisted after the fourth-day after surgery. These biochemical manifestations are compatible with hungry bone syndrome (HBS). Despite calcitriol prophylaxis, the patient developed HBS; therefore, a calcium infusion was initiated. In a prospective randomized study with 102 patients, there was no statistically significant benefit of using vitamin D (cholecalciferol) in patients with vitamin D deficiency to reduce HBS incidence after parathyroidectomy; this study demonstrated a raised incidence of HBS in younger individuals [16]. It has been postulated that AP is the best HBS predictor after parathyroidectomy [17]. In this case, values of AP oscillated between 600 and 2000 UI/L before surgery. Recently, it has been proposed that double adenomas are a different entity from multiglandular hyperplasia. Histopathological and molecular criteria are not well defined to distinguish between these two pathologies, however, the strongest criterion that supports the diagnosis of double adenomas is the absence of recurrence of hypercalcemia in at least 5 years after subtotal parathyroidectomy [18]. Principal limitations in this case report were the inaccessibility of studies such as sestamibi gammagraphy for hyperactive hyperparathyroid glands, nonetheless, high-definition ultrasonography remains the mainstay for preoperative location, and when combined with other study modalities such as a CT scan, the intraoperative identification rate can be enhanced [19]. Another limitation was the lack of availability to measure PTH levels after subtotal parathyroidectomy, nonetheless, because of the HBS presence in this patient, indirectly, we concluded there was suppression in PTH secretion.

## Conclusions

The diagnosis of double parathyroid adenomas is difficult. Regarding the similarities between multiglandular hyperplasia and parathyroid adenomas, this case report contributes to the further distinction between these two clinical entities. This case report represents, in particular, the challenge of a difficult diagnosis, due to the limited available resources.

## Data Availability

The data and materials/figures used in the current study are available from the corresponding author upon reasonable request.

## Abbreviations

PHPT: Primary hyperparathyroidism
CT: Computed tomography
PTH: Parathyroid hormone
MEN 1/2: Multiple endocrine neoplasia
HBS: Hungry bone syndrome
GPAs: Giant parathyroid adenomas
AP: Alkaline phosphatase
3D: Third dimension
LVH: Left ventricular hypertrophy
